# Advances in Functional Rubber and Elastomer Composites II

**DOI:** 10.3390/polym17162247

**Published:** 2025-08-20

**Authors:** Vineet Kumar, Md Najib Alam

**Affiliations:** School of Mechanical Engineering, Yeungnam University, 280, Daehak-ro, Gyeongsan 38541, Republic of Korea; vineetfri@gmail.com

## 1. Introduction

Recently, rubber and elastomer materials have expanded their applications from traditional mechanical uses to advanced mechanical, electrical, and sensor applications [1]. To achieve these goals, functionalization of both the rubber matrix and the fillers is often necessary. For example, rubber is a good dielectric material, and incorporating electrically conductive fillers significantly enhances its dielectric properties, making it useful for nanogenerators, actuators, and sensor applications [2,3,4,5]. Similarly, some advanced elastomer composites, such as magnetorheological elastomers, have smart mechanical applications because their properties can be tuned by an external magnetic field [6,7]. However, in many rubber composite applications, the fillers used are sometimes synthetic and non-renewable [8,9,10]. From environmental and health perspectives, rubber scientists are striving to address these issues by developing eco-friendly alternatives [8,9,10].

The previous related Special Issue, “Advances in Functional Rubber and Elastomer Composites,” discussed developments in rubber and polymer composites with enhanced mechanical, thermal, dielectric, and functional properties [11,12,13,14,15,16,17,18,19,20,21,22,23,24,25,26,27,28]. This Special Issue focuses on further advancements in mechanical, magnetomechanical, electrical, energy harvesting, and sensing properties, critically examining the roles of rubber and functional filler materials in these composites.

## 2. Overview of Published Articles

Dai et al. [29] proposed a novel alkaline protease/calcium chloride coagulation method that enhances natural rubber’s mechanical properties while reducing environmental impact. Compared to acid coagulation, this new method improves strength, abrasion resistance, and thermal stability, while minimizing soil acidification—enabling eco-friendly, high-performance rubber without chemical modification. Torres et al. [30] created sustainable composites with enhanced mechanical and thermal properties by combining bovine leather waste with natural rubber (TSR-20). The resulting material shows strong rubber–leather interaction, high density, and PVC-like hardness, offering eco-friendly applications in footwear and fashion, while promoting waste valorization and resource recycling. Alam et al. [31] compared various properties of CNT-reinforced natural rubber (NR) and nitrile butadiene rubber (NBR) composites. NR shows greater mechanical improvement due to stronger CNT bonding, while NBR exhibits superior piezoresistive sensitivity and electromechanical actuation. NR is suitable for dynamic applications; NBR excels in stretchable sensors and actuators due to its polar functionality. Luengchavanon et al. [32] synthesized Ag_mgt_/Zn_mgt_ nanocomposites using mangosteen peel extract, which were applied to rubber gloves and showed strong antibacterial activity, including against MRSA, with MICs of 40–320 µg/mL. Despite effective bacterial inhibition in suspensions, glove coatings required at least 30 min of contact time for antimicrobial efficacy. Yu et al. [33] studied three 5% fiber-reinforced TPU composites for water-lubricated bearings. Basalt fiber showed the best tribological performance, reducing friction by 70.57% and wear by 98.69% compared to pure TPU. UHMWPE and bamboo fibers performed well under high loads, enhancing surface smoothness and wear resistance. Ding et al. [34] examined how different coagulation methods affect the hyperelastic behavior of natural rubber using tensile tests and an improved Yeoh model. Results reveal distinct mechanical properties across methods, highlighting the importance of coagulation in rubber performance and offering insights for optimizing natural rubber processing. Li et al. [35] presented a simplified GRU-attention-based constitutive model for high-damping rubber (HDR), implemented as HDRGA material in OpenSees. Validation using structural models shows the HDRGA material accurately simulates HDR’s seismic behavior, offering a reliable tool for earthquake-resistant design in civil engineering. Beknazarov et al. [36] evaluated shungite ore and its concentrate as partial replacements for carbon black in nitrile butadiene rubber. Flotation increased carbon content and improved tensile strength, reduced viscosity, and extended curing times without affecting oil resistance, demonstrating shungite’s potential as an alternative rubber filler. Yangthong et al. [37] studied epoxidized natural rubber (ENR) composites filled with alumina, silica, and hybrid fillers, showing enhanced thermal and mechanical properties. The ENR/hybrid (25/25 phr) filler achieved superior conductivity (2.23 W/mK) and strength. This cost-effective alternative to silicone rubber is promising for electronics, batteries, and energy-related applications. Ge et al. [38] studied a self-oscillating liquid crystal elastomer (LCE) fiber-slide system driven by a self-flickering light source under constant voltage. The design enables synchronized energy input, enhancing efficiency and simplicity. It offers potential applications in dynamic circuits, sensing systems, and optical devices due to its autonomous and periodic motion. Inphonlek et al. [39] investigated enhanced deproteinized natural rubber by grafting acrylic acid and acrylamide, enabling coordination with silver and titanium dioxide. The resulting MDPNR/Ag-TiO_2_ composites showed improved dielectric properties, thermal stability, stiffness, and antibacterial activity, with well-dispersed silver and increased dielectric constant compared to unmodified rubber. Akkenzheyeva et al. [40] fabricated the rubber–polymer composite ELTC, derived from devulcanized tires and plastics, which was used to modify asphalt binders. ELTC improved binder homogeneity, thermal stability, softening temperature, and resistance to summer plastic deformation. Its even distribution and good component compatibility enhanced the performance of the modified asphalt binders. Wang et al. [41] examined how free and bonded proteins affect natural rubber’s vulcanized network and mechanical properties. Reduced protein content decreased entanglement, crosslink density, and strength. Bonded proteins influenced vulcanization, while free proteins affected crosslink density. Both protein types contribute to stronger, more robust vulcanized rubber. Glavan et al. [42] investigated the dynamic strain response of soft magnetoactive elastomers (MAEs) under alternating magnetic fields. MAEs exhibit significantly higher piezomagnetic strain coefficients than conventional magnetostrictive composites, with notable phase lag. The results highlight nonlinear viscoelasticity as a key factor influencing MAE actuator performance in soft robotics. Bao et al. [43] synthesized siloxane polyurea copolymer elastomers using low and high Mn monomers to study structure–property relationships. Film analyses revealed that tensile properties, transparency, Tg, and elasticity depend on composition. Hydrogen bonding strength and recovery, assessed via IR and tensile tests, correlate with hard segment Mn and influence macroscopic performance. Alam et al. [44] developed cost-effective, multifunctional styrene–butadiene rubber composites using iron, aluminum, and hybrid metal fillers. Iron enhances electrical and magnetic properties but reduces crosslink density, while aluminum improves modulus. At 20 vol%, iron-filled composites show strong magnetic anisotropy and conductivity, suggesting potential for smart applications with future reinforcement improvements. Liu et al. [45] synthesized three reactive POSS modifiers to toughen epoxy resins without compromising key properties. The resulting hybrid resins showed good compatibility, low viscosity, low curing temperature, and significantly improved toughness. Notably, OGCPS-EP-0.6-C achieved a 58.75% increase in KIC with minimal changes in glass transition temperature and flexural strength. Abdollahi et al. [46] developed a 3D finite element model using a microscale representative volume element (RVE) to predict the shear behavior of magnetorheological elastomers (MREs) under magnetic fields. Incorporating different silicone matrices and carbonyl iron particles, the model accurately captures field-dependent responses, offering insights for adaptive vibration absorber design. Magaletti et al. [47] replaced silica with SP-functionalized carbon black (CB/SP) in elastomer composites for tire applications. Using TESPT as a coupling agent, CB/SP-based composites show lower hysteresis, reduced Payne effect, and higher dynamic rigidity. While slightly lower in ultimate properties, they offer environmental benefits and reduced weight compared to silica-based systems. Alam et al. [48] showed that adding 1–2 phr MgO to ZnO-based sulfur vulcanization systems significantly accelerates curing kinetics. MgO reduces vulcanization time and allows curing at lower temperatures (down to 100 °C), maintaining or improving mechanical properties. This approach offers a more energy-efficient, cost-effective, and eco-friendly rubber vulcanization process. Leyva-Porras et al. [49] used swelling and mechanical tests with the Flory–Rehner equation to estimate the Flory–Huggins interaction parameter (χ12*) in crosslinked elastomer blends of BR, SBR, and NBR. Results indicate preferential crosslinking within phases, blend immiscibility, and slightly higher χ12* values than literature reports. Kitsawat et al. [50] developed green natural rubber composites reinforced with up to 60 phr synthetic graphite platelets using alginate, showing significantly enhanced mechanical strength, chemical resistance, hydrophilicity, and electrical conductivity. The composites maintained biodegradability (21–30% after 90 days) and demonstrated potential for electronic substrate applications without agglomeration issues. Kodal et al. [51] demonstrated that adding multi-walled carbon nanotubes (MWCNTs) to carbon black-reinforced NR/SBR tire compounds improves filler dispersion, vulcanization, mechanical properties, and thermal conductivity. Enhanced heat dissipation reduces heat build-up, with a percolation model explaining the synergistic effects of hybrid carbon fillers in the composites. Rebane et al. [52] compared hydroxyl-bearing blowing agents in silica- and mica-filled silicone foams, achieving low-density, uniform porosity, and enhanced mechanical properties. Adjusting diol chain length and combining agents like glycerol with water reduced foam density by up to 30% while improving tensile strength, with fast curing and suitable viscosity for industrial processing. Zhao et al. [53], in a review, explored wet mixing technology for dispersing fillers like graphene, carbon nanotubes, silica, and carbon black in natural rubber (NR) composites. The review highlights improved filler dispersion and reinforcement mechanisms, discusses preparation techniques, and identifies current limitations and challenges to guide future advancements in wet mixing methods.

## 3. Summary and Future Outlook

This collection of recent research highlights major advances in sustainable and high-performance rubber and elastomer composites. A novel alkaline protease and calcium chloride coagulation method has been developed to improve natural rubber’s mechanical properties while reducing environmental impact. Sustainable composites using bovine leather waste in natural rubber have shown enhanced mechanical and thermal properties, making them suitable for eco-friendly applications. Comparisons between CNT-reinforced natural rubber and nitrile butadiene rubber reveal that natural rubber offers better mechanical reinforcement, while nitrile rubber demonstrates superior electromechanical performance for sensing and actuation.

Antibacterial nanocomposites synthesized using mangosteen peel extract have shown promising applications in rubber gloves. Fiber-reinforced thermoplastic polyurethane composites, especially those with basalt fiber, exhibit significant improvements in friction and wear resistance for water-lubricated bearings. Studies on coagulation methods indicate their significant impact on the hyperelastic behavior of natural rubber. A new GRU-attention-based model accurately simulates the seismic response of high-damping rubber, aiding structural design.

Further innovations include using shungite ore as a filler alternative, hybrid fillers in epoxidized rubber for improved thermal conductivity, and self-oscillating liquid crystal elastomer systems for energy-efficient motion. Modified deproteinized rubber with metal oxides enhances dielectric and antibacterial properties. Recycled rubber–plastic composites improve asphalt performance. Protein content in rubber has been shown to influence crosslinking and mechanical strength.

Magnetoactive elastomers exhibit enhanced piezomagnetic responses under magnetic fields. Siloxane polyurea copolymers demonstrate structure–property relationships depending on hydrogen bonding. Hybrid metal fillers in styrene–butadiene rubber enable multifunctional performance, and POSS modifiers toughen epoxy resins without sacrificing key characteristics. Additional advancements include predictive models for magnetorheological elastomers, functional carbon black for tire applications, energy-efficient vulcanization using MgO, and biodegradable natural rubber composites with synthetic graphite. Wet mixing technology continues to evolve, offering improved filler dispersion and reinforcing effects in rubber composites.

Despite significant advancements, challenges still remain in achieving the required mechanical, electrical, and thermal properties for rubber composites to meet industrial application standards. Therefore, addressing these issues will be essential in future research to fully realize their practical potential.
